# Right-handed Z-DNA at ultrahigh resolution: a tale of two hands and the power of the crystallographic method

**DOI:** 10.1107/S2059798322011937

**Published:** 2023-01-20

**Authors:** Pawel Drozdzal, Tomasz Manszewski, Miroslaw Gilski, Krzysztof Brzezinski, Mariusz Jaskolski

**Affiliations:** a Institute of Bioorganic Chemistry, Polish Academy of Sciences, Poznan, Poland; b Department of Crystallography, Faculty of Chemistry, Adam Mickiewicz University, Poznan, Poland; University of Virginia, USA

**Keywords:** Z-DNA, handedness, anomalous signal, 2′-deoxy-l-ribose, dual-conformation backbone, cadaverinium cation, biogenic polyamines, biological potassium complex

## Abstract

The self-complementary l-d(CGCGCG)_2_ purine/pyrimidine hexanucleotide was crystallized in complex with the polyamine cadaverine and potassium cations. Since the oligonucleotide contained the enantiomeric 2′-deoxy-l-ribose, the Z-DNA duplex is right-handed, as confirmed by the ultrahigh-resolution crystal structure determined at 0.69 Å resolution. Although the X-ray diffraction data were collected at a very short wavelength (0.7085 Å), where the anomalous signal of the P and K atoms is very weak, the signal was sufficiently outstanding to clearly indicate the wrong hand when the structure was mistakenly solved assuming the presence of 2′-deoxy-d-ribose. The electron density clearly shows the entire cadaverinium dication, which has an occupancy of 0.53 and interacts with one Z-DNA duplex. The K^+^ cation, with an occupancy of 0.32, has an irregular coordination sphere that is formed by three OP atoms of two symmetry-related Z-DNA duplexes and one O5′ hydroxyl O atom, and is completed by three water sites, one of which is twofold disordered. The K^+^ site is complemented by a partial water molecule, the hydrogen bonds of which have the same lengths as the K—O bonds. The sugar-phosphate backbone assumes two conformations, but the base pairs do not show any sign of disorder.

## Abstract 2

The crystal structure of the self-complementary d(CGCGCG)_2_ Z-DNA duplex in complex with cadaverinium and potassium cations was solved at ultrahigh resolution. The oligonucleotide used for crystallization contained the enantiomeric 2′-deoxy-l-ribose instead of its natural d-enantiomer, and thus the Z-DNA duplex is right-handed.

## 1. Introduction

Natural d-deoxyoligonucleotides [for example d-d(CGC­GCG)_2_; d-DNA] and their synthetic enantiomers [l-d(CGC­GCG)_2_, l-DNA] with the same sequence have the same physical (for example duplex stability or solubility) and chemical properties (Hauser *et al.*, 2006 ▸). However, the l-enantiomers do not interact with biological partners and, not surprisingly, they cannot serve as templates for DNA or RNA polymerases (Hayashi *et al.*, 2005 ▸; Hauser *et al.*, 2006 ▸). The l-enantiomers are also resistant to nuclease degradation and to many off-target interactions that plague traditional d-oligonucleotide-based technologies (Anderson *et al.*, 1984 ▸; Asseline *et al.*, 1991 ▸; Damha *et al.*, 1994 ▸; Williams *et al.*, 1997 ▸; Hauser *et al.*, 2006 ▸). This makes them ideal for biomedical applications (for example as aptamers or biosensors), as well as attractive objects for molecular biology (for example for chiral separations) or DNA nanotechnology (Young *et al.*, 2019 ▸).

It has been shown that polyamines are able to induce secondary-structure transitions of DNA, with the B–Z transition of alternating purine/pyrimidine oligonucleotides in solution being particularly enhanced by polyamines (van Dam *et al.*, 2002 ▸). Cadaverine^2+^ [Cad^2+^; 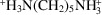 ], a biogenic amine, is a positively charged organic dication under physiological ionic and pH conditions and hence can interact with negatively charged macromolecules such as DNA and RNA. In solution, 1,3-diaminopropane (DAP) and diamino­ethane are similarly effective in the B–Z conversion, with both being slightly more effective than putrescine^2+^ [Put^2+^; 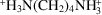 ] and more effective than Cad^2+^ (Behe & Felsenfeld, 1981 ▸). In another study, thermodynamic analysis of the interactions of biogenic polyamines with genomic DNA showed that polyamines bound more strongly to AT-rich DNA compared with DNA with a high GC content and that the binding efficiency varied depending on the charge of the polyamine as follows: spermine^4+^ > spermidine^3+^ > putre­scine^2+^ > cadaverine^2+^ (Kabir & Kumar, 2013 ▸). However, the Cad^2+^ ion is specified as a ligand in only a few protein structures deposited in the PDB (Berman *et al.*, 2000 ▸). Thus, Cad^2+^ is very poorly characterized in the context of its interactions with nucleic acids.

In this paper, we describe an ultrahigh-resolution crystal structure of Z-DNA with the sequence l-d(CGCGCG)_2_, in which the natural 2′-deoxy-d-ribose was replaced with its l-enantiomer. This is the first crystal structure determination of right-handed Z-DNA, with the caveat that the right-handed enantiomer was also present, together with the natural left-handed duplex, in the centrosymmetric crystal structure of the same DNA (Doi *et al.*, 1993 ▸; Drozdzal *et al.*, 2016 ▸).

The story behind this project is very interesting and demonstrates the power and sensitivity of the crystallographic method. The postdoc who was synthesizing the d(CGCGCG)_2_ duplex for a crystallization experiment with cadaverine^2+^ ran out of the 2′-deoxy-d-ribose oligonucleotide, and thinking (basically correctly, if handedness is neglected) that it would not matter very much, used for this purpose the 2′-deoxy-l-ribose oligonucleotide at hand, a leftover from a ‘centro­symmetric project’ (Drozdzal *et al.*, 2016 ▸). He grew excellent crystals and then left. The ultrahigh-resolution X-ray diffraction data, obviously collected at a very short wavelength (0.7085 Å), were inherited by another postdoc, who was unaware of the swap of hands in the crystallization experiment. He was extremely puzzled when his high-resolution refinements kept telling him that the model was wrong and that the structure enantiomorph should be inverted. This indication was given by the Flack and Pearson tests (Flack, 1983 ▸; Flack & Bernardinelli, 2008 ▸), as included in the *SHELXL* (Sheldrick, 2015 ▸) software, and also by the Hooft test (Hooft *et al.*, 2008 ▸) as implemented in *PLATON* (Spek, 2009 ▸). After switching the structure enantiomorph the refinement was unproblematic, but the Z-DNA duplex became right-handed. The first postdoc was then contacted and he explained his experiment. At this point everything started making sense again.

In addition to describing an ultrahigh-resolution structure of a right-handed Z-DNA duplex, this paper also provides, for the first time, insight into the interaction of (Z-)DNA with the cadaverinium dication (Cad^2+^). After inspection of all of the crystal structures of Z-form DNA in the PDB as well as those described in the scientific literature, we compiled a list of 68 nucleic acid structures in complex with polyamines and/or metal cations (Supplementary Table S1). Among these crystal structures, there are 44 Z-DNA/metal^*m*+^ complexes, 16 Z-DNA/polyamine^*n*+^/metal^*m*+^ and eight Z-DNA/polyamine^*n*+^ complexes, none them with cadaverine.

## 2. Materials and methods

### 2.1. Oligonucleotide synthesis, purification and crystallization

The methods for the synthesis, deprotection and purification of the oligodeoxynucleotide have been described previously (Xia *et al.*, 1998 ▸; Drozdzal *et al.*, 2013 ▸). A 1.5 m*M* water solution of the 2′-deoxy-l-ribose-containing DNA oligonucleotide with the self-complementary sequence l-d(CGCGCG) was heated at 338 K for 10 min and then slowly annealed to room temperature overnight. Single crystals of l-d(CGCGCG)_2_/Cad^2+^/K^+^ were grown at 292 K by the hanging-drop vapor-diffusion method by mixing 2 µl nucleic acid solution and 2 µl precipitating solution consisting of 10%(*v*/*v*) (±)-2-methyl-2,4-pentanediol (MPD), 40 m*M* sodium cacodylate pH 6.0, 80 m*M* KCl, 12 m*M* NaCl, 14 m*M* cadaverinium dichloride. The drops were equilibrated against 0.5 ml 80%(*v*/*v*) MPD. Crystals appeared within one week and grew to dimensions of 0.3 × 0.1 × 0.1 mm.

### 2.2. X-ray data collection and processing

X-ray diffraction data for the l-d(CGCGCG)_2_/Cad^2+^/K^+^ complex were measured to 0.69 Å resolution on the EMBL P13 beamline at the PETRA III synchrotron at DESY, Hamburg. The crystal was vitrified in a stream of cold nitrogen gas at 100 K. The mother liquor served as a cryoprotectant. The diffraction data were collected at a wavelength of 0.7085 Å and were indexed, integrated and scaled using the *XDS* package (Kabsch, 2010 ▸), as summarized in Table 1 ▸.

### 2.3. Structure solution and refinement

The structure was solved by molecular replacement using *Phaser* (McCoy *et al.*, 2007 ▸). The postdoc performing the structure-solution step was unaware that the crystal contained the l-d(CGCGCG)_2_ ‘spiegelmer’ of the oligonucleotide and he used, quite naturally, the DNA part of PDB entry 7atg, corresponding to our earlier model of the d-d(CGCGCG)_2_/Put^2+^/K^+^ complex (Drozdzal *et al.*, 2021 ▸), as the molecular probe. Since Friedel’s law makes the diffraction pattern centrosymmetric (with the exception of the, usually small, deviations caused by anomalous scattering), a noncentro­symmetric crystal structure can be solved equally well, of course, by both enantiomers of the molecular model.

In the initial stages of the refinement, the model was refined using *REFMAC*5 (Murshudov *et al.*, 2011 ▸) from the *CCP*4 suite (Winn *et al.*, 2011 ▸). The final anisotropic refinement was carried out with *SHELXL* (Sheldrick, 2015 ▸) using the full resolution of the diffraction data. The details of the *SHELXL* refinement were the same as described for our previous Z-DNA structures (Drozdzal *et al.*, 2013 ▸, 2015 ▸). At this resolution, no stereochemical restraints are necessary to supplement the experimental observations (Jaskolski, 2017 ▸). However, restraints may still be needed for some disordered or highly mobile fragments. In the present structure, restraints were only applied to the cadaverinium dication and to the bonds and angles of dual-conformation Z-DNA fragments. The ideal geometry targets for Cad^2+^ were taken from a high-quality X-ray structure of cadaverinium dichloride (Pospieszna-Markiewicz *et al.*, 2006 ▸). Conformation-dependent geometrical restraints on bond lengths (DFIX) and bond angles (DANG) for the polynucleotide chains were generated using the *RestraintLib* server (http://achesym.ibch.poznan.pl/restraintlib/) as described by Kowiel *et al.* (2016 ▸, 2020 ▸) and Gilski *et al.* (2019 ▸). The CSD-derived conformation-dependent *RestraintLib* dictionary supersedes the classic nucleic acid restraints compiled by Clowney *et al.* (1996 ▸), Gelbin *et al.* (1996 ▸) and Parkinson *et al.* (1996 ▸). The final cycles of CGLS (conjugate-gradient least-squares) refinement converged with an *R* and *R*_free_ of 10.32% and 12.83%, respectively. The very last round of refinement, calculated with the test reflections included in the working set, converged with *R* = 10.39%. In order to provide estimations of standard uncertainties in all individual refined parameters and all derived geometrical parameters, in the final stage of the refinement one cycle of full-matrix least-squares minimization was calculated. The placement of the model in the unit cell was standardized using the *ACHESYM* server (Kowiel *et al.*, 2014 ▸).

At the wavelength used in the diffraction experiment (0.7085 Å), the imaginary components of the anomalous scattering (*f*′′) of K and P atoms are 0.252 and 0.098 electron units, respectively (Cromer, 1983 ▸). Anomalous signal is visible in the diffraction data up to ∼0.9 Å resolution and therefore the refinement was carried out against unmerged anomalous data. The signal was quite weak, however, as no clear peaks were located in the anomalous electron-density map contoured at the 3σ level that could correspond to the positions of the K^+^ ion and P atoms.

*Coot* (Emsley *et al.*, 2010 ▸) was used for visualization of the electron-density maps and manual rebuilding of the atomic model.

### 2.4. Determination of the absolute configuration of the d(CGCGCG)_2_ DNA hexamer

Intriguingly, at this stage the structure refinement performed with *SHELXL* (Sheldrick, 2015 ▸) indicated an unexplained issue with model chirality. The Flack and Pearson parameters (Flack, 1983 ▸; Flack & Bernardinelli, 2008 ▸) calculated by the *SHELXL* program were 0.90 (8) and 0.95 (3), respectively. Moreover, model validation performed with *PLATON* (Spek, 2009 ▸) gave a similar warning, as the Flack, Pearson and Hooft (Hooft *et al.*, 2008 ▸) parameters were 0.95 (3), 0.80 (2) and 0.89 (2), respectively. These results attracted our attention, as they obviously indicated the wrong configuration of the refined model. When it became clear that 2′-deoxy-l-ribose derivatives had been used instead of their natural d-counterparts in the synthesis of the deoxy­oligonucleotide, the absolute configuration of the structure was inverted through the use of the MOVE command in *SHELXL*. When the inverted model was checked again with *PLATON*, the Flack, Pearson and Hooft parameters were calculated to be 0.03 (3), 0.12 (2) and 0.08 (2), respectively, clearly indicating the correct handedness, which corresponds to the right-handed l-d(CGCGCG)_2_ DNA duplex. Also, the Flack and Pearson parameters calculated by the *SHELXL* program [0.03 (8) and 0.03 (3), respectively] confirmed the correct chirality of the new model. Based on the final and correct model, the 3*DNA* program (Lu & Olson, 2003 ▸) was used to calculate the geometrical parameters of the l-DNA molecule. Figures illustrating the atomic models and the electron density were prepared with *PyMOL* (DeLano, 2002 ▸).

## 3. Results

### 3.1. Quality of the results

The estimated standard uncertainty (e.s.u.) values of fully occupied DNA atomic positions in the l-d(CGCGCG)_2_/Cad^2+^/K^+^ structure are in the ranges 0.006–0.052 Å for C atoms, 0.005–0.009 Å for N atoms, 0.006–0.101 Å for O atoms and 0.002–0.003 Å for P atoms. The positional uncertainty of the K^+^ cation is 0.021 Å. The e.s.u. values for full-occupancy covalent bonds are ∼0.006, ∼0.006, ∼0.005 and ∼0.004 Å for C—C, C—O, C—N and P—O, respectively. The r.m.s.d. agreement with stereochemical standards is 0.012 Å for bond lengths and 2.10° for bond angles.

### 3.2. Overall structure and helical parameters

**Figure 1:**
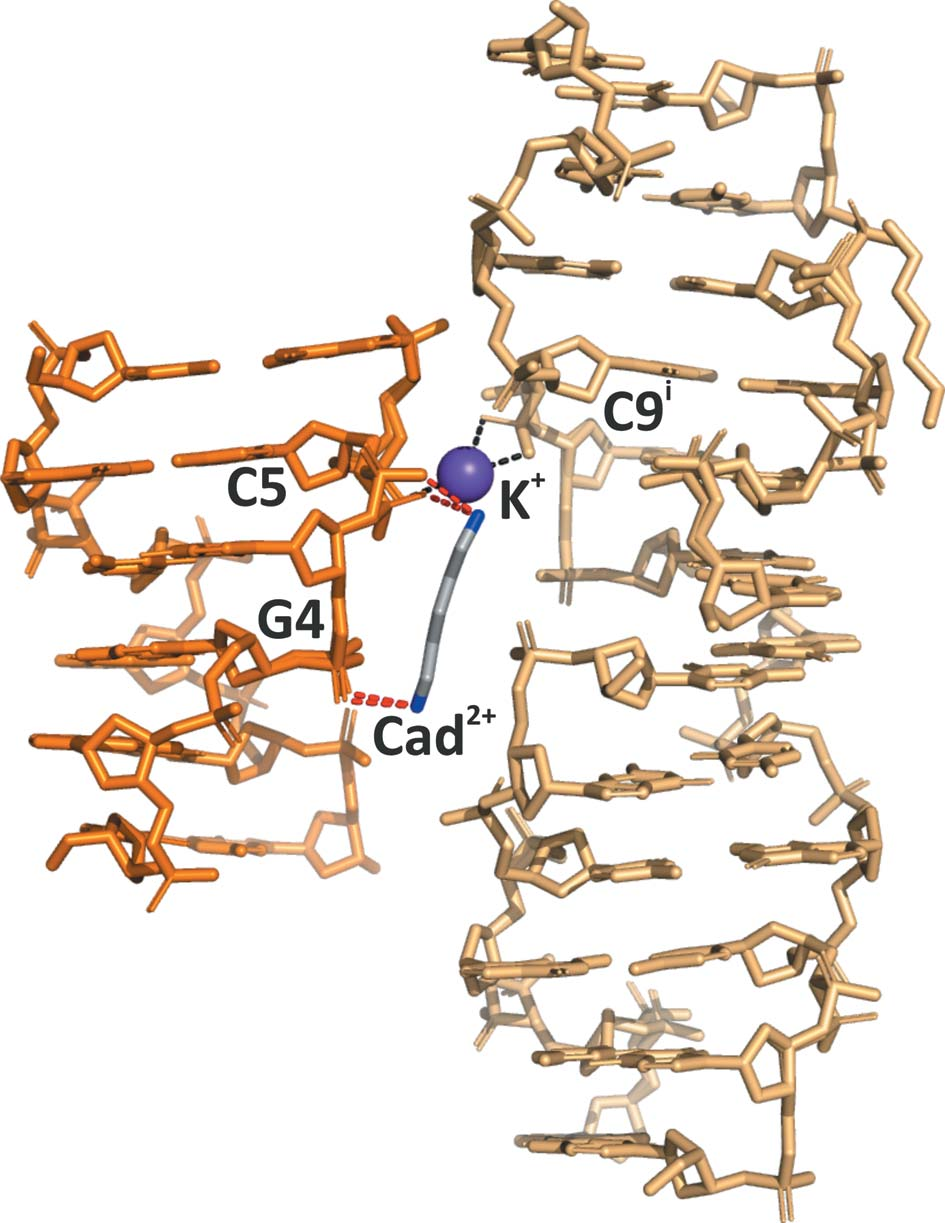
Overall structure of l-d(CGCGCG)_2_/Cad^2+^/K^+^ highlighting the inter­actions between the cations and the DNA molecules within the asymmetric unit (orange) and two other symmetry-related DNA molecules (light orange): i (−*x*, *y* + 1/2, −*z* + 1/2) and ii (*x* + 1/2, −*y* + 1/2, −*z*). Potential polar interactions with the Cad^2+^ cation (stick model) are marked as red dashed lines and K^+^ is shown as a purple sphere with the coordination bonds marked as black dashed lines.

The l-d(5′-CGCGCG-3′) nucleosides of the l-d(CGCGCG)_2_ duplex in the asymmetric unit (Fig. 1 ▸) are numbered from 1 to 6 in chain *A* and from 12 to 7 in the complementary l-d(3′-GCGCGC-5′) strand *B*. The base-pair scheme is C1·G12, G2·C11, …, G6·C7. There are only five internucleoside phosphate linkages in each CGCGCG strand, as there are no phosphate groups at the 5′- and 3′-ends. Within the DNA molecule, two alternative conformations (labeled I and II) are clearly visible in the electron-density map at the inter-nucleotide phosphate linkages between C3–G4, G4–C5, C5–G6 and G8–C9, the refined occupancies of which converged at 0.575 (21)/0.425 (22), 0.556 (6)/0.444 (6), 0.597 (13)/0.403 (13) and 0.80 (12)/0.20 (12), respectively. Moreover, the *mF*_o_ − *DF*_c_ electron-density map indicated one additional discrete position (peak height ∼6.5σ) for the P atom of the C3 nucleotide. However, an additional phosphate group placed at this peak refined with an occupancy below 0.20. Therefore, no alternative conformer was modeled to interpret this peak. No alternative conformations were observed for the base moieties.

The geometry of the duplex agrees well with the expected stereochemistry and with other crystallographic models of Z-DNA, including the ultrahigh-resolution PDB entry 3p4j (Supplementary Table S2) determined at 0.55 Å resolution (Brzezinski *et al.*, 2011 ▸). The pseudorotation of the deoxy­ribose moieties, analyzed according to the method of Jaskólski (1984 ▸), is typical for Z-DNA models and corresponds to the C2′-*endo*/C3′-*endo* pucker of the pyrimidine/purine 3′,5′-nucleotides. Also in the present complex, the sugars at the 3′-termini do not have the alternating C2′-*endo*/C3′-*endo* pucker for the pyrimidine/purine nucleotides, as is typical for Z-DNA, but all assume the C2′-*endo* conformation. There are two conformational subforms of Z-DNA, namely ZI and ZII, differing in the torsion angles α [defined as O3′(*i* − 1)—P—O5′—C5′] and ζ [C3′—O3′—P(*i* + 1)—O5′(*i* + 1)] (Saenger, 1984 ▸), stabilized by hydrogen bonding of a phosphate group to a hydrated metal cation or water molecule(s). Here, the ZII conformation of the phosphate group can only be assigned to G4(I), with ζ = 62.7° (the signed ζ torsion angle is given for the reference 2′-deoxy-d-ribose enantiomer). This alternative ZII conformer is stabilized by hydrogen bonds to the N1 atom of Cad^2+^ and a water molecule. The remaining phosphate groups have the ZI conformation.

### 3.3. Binding of the cadaverinium dication

**Figure 2:**
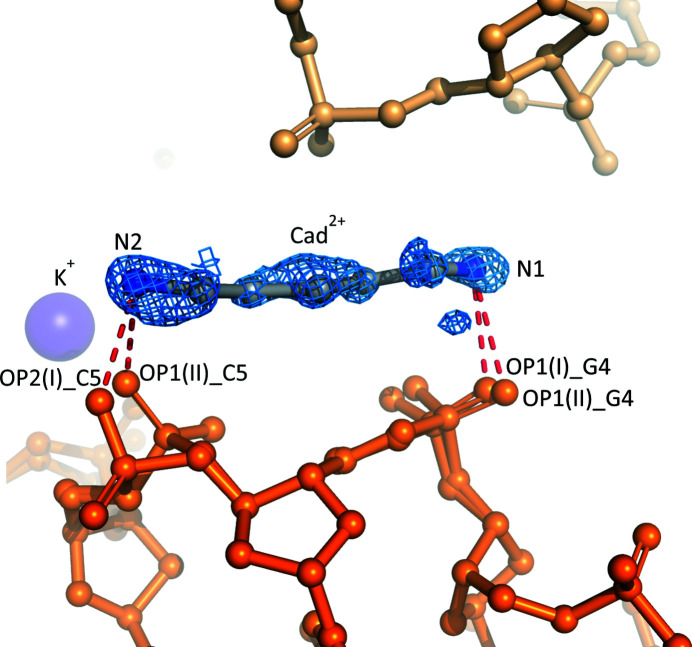
Details of the binding mode of the cadaverinium dication within the crystal lattice of the l-d(CGCGCG)_2_/Cad^2+^/K^+^ complex. The 2*mF*_o_ − *DF*_c_ map (blue) is contoured at the 1.0σ level. The color code for molecules and ions is the same as in Fig. 1 ▸.

The entire cadaverinium dication is well defined in the electron-density map (Fig. 2 ▸) despite its partial occupancy of 0.526 (13). It has the all-*trans* conformation, with the following torsion angles: 170 (1), −173 (1), 172 (1) and 175 (2)°. The Cad^2+^ dication is only involved in hydrogen-bonding inter­actions with the O atoms of the phosphate groups of one DNA molecule (Figs. 1 ▸ and 2 ▸). The N1 atom of the Cad^2+^ dication forms hydrogen bonds to phosphate groups of the major (I) and minor (II) conformer. These include interactions with OP1(I)_G4 and OP1(II)_G4 at distances of 2.776 (22) and 2.833 (34) Å, respectively. The N2 atom is hydrogen-bonded to OP2(I)_C5 and OP1(II)_C5 at 2.760 (15) and 2.842 (15) Å, respectively. Additionally, the Cad^2+^ ion forms hydrogen bonds to water molecules, which in turn interact with other phosphate groups of two symmetry-related molecules, indicated as i (−*x*, *y* + 1/2, −*z* + 1/2) and ii (*x* + 1/2, −*y* + 1/2, −*z*). It is of note that the Cad^2+^ cation does not form any direct hydrogen bonds to symmetry-related Z-DNA molecules. We point out that since cadaverine is an achiral molecule, its interactions with both enantiomers of the Z-DNA molecule will be the same.

### 3.4. Coordination of the K^+^ cation

**Figure 3:**
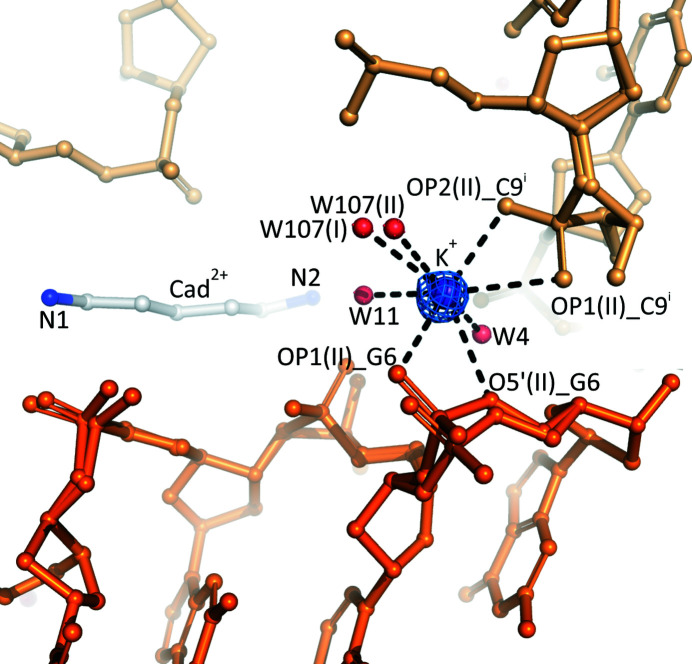
Potassium (purple sphere) coordination site in the crystal structure of the l-d(CGCGCG)_2_/Cad^2+^/K^+^ complex. Water molecules (W) are presented as red spheres. The coordination sphere of this intermolecular potassium binding site is completed by partially disordered phosphate groups from two symmetry-related DNA molecules (orange and light orange). The 2*mF*_o_ − *DF*_c_ map (blue) is contoured at the 1.0σ level.

The electron-density maps clearly revealed one metal coordination site, initially interpreted as K^+^ (Fig. 3 ▸). Since the occupancy of this monovalent cation refined to a fractional value of 0.553 (9), a complementary water molecule was also modeled in the 2*mF*_o_ − *DF*_c_ map at this site. After refinement of this model, the occupancies of K^+^ and the water molecule were 0.318 (17) and 0.682 (17), respectively. There are seven O atoms in the immediate vicinity of this site, four from the Z-DNA backbone (three OP atoms of two symmetry-related Z-DNA duplexes and one O5′ hydroxyl O atom) and three from water molecules W4, W11 and W107 (the latter water molecule is partially disordered and has been modeled in two distinct positions). As in the previously studied Z-DNA/Put^2+^/K^+^ complex (Drozdzal *et al.*, 2021 ▸), the K^+^ cation in the present structure is also located between two Z-DNA phosphate groups [OP1(II)_G6 and OP1/2(II)_C9^i^]. The K^+^—O bond distances are in the range 2.491 (12) to 3.017 (33) Å. The lengths of the *M* ^+^—O bonds support the presence of K^+^ rather than the presence of Na^+^ at higher occupancy. The *CheckMyMetal* server (Zheng *et al.*, 2014 ▸) also predicted potassium as the most likely cation at this site. The coordination sphere (coordination number seven) can be considered as distorted pentagonal bipyramid or a capped octahedron. The angles within the coordination sphere are irregular (Table 2 ▸).

### 3.5. Solvent structure and hydration of the right-handed Z-DNA helix

The crystallographic model presented in this work is similar to other high-resolution Z-DNA structures, also from the point of view of the architecture of the hydration shells (Drozdzal *et al.*, 2013 ▸, 2015 ▸, 2021 ▸). Specifically, the asymmetric unit contains complicated hydrogen-bonded networks involved in crystal packing and stabilization of the conformation of the oligonucleotides. Not surprisingly, there are no significant differences in the hydration of the enantiomeric forms of Z-DNA. The asymmetric unit contains 121 water sites, which were refined anisotropically without positional restraints. There was no attempt to model the H atoms of the water molecules. There are 29 close pairs of water molecules with a combined occupancy of 1.0. The remaining water sites were classified as fully (25) or partially (67) occupied. Summation of all the water occupancies in the asymmetric unit gives a total of 90.30 water molecules. It should be noted that our final model (DNA + solvent region) generates a problem of electrostatic neutrality of the crystal structure. The ten negatively charged phosphate groups are only partially neutralized by the two protonated amine groups of the partially occupied Cad^2+^ dication and by the fractional potassium cation. Our attempts to identify other positively charged species within the asymmetric unit that would ensure electrostatic neutrality were unsuccessful. A similar problem was also unresolved even for the ultrahigh-resolution Z-DNA structure at 0.55 Å resolution (Brzezinski *et al.*, 2011 ▸).

## 4. Discussion

In this work, we have presented a new crystal structure of right-handed Z-DNA in complex with cadaverine^2+^ and K^+^ cations. It is the first example of interaction of cadaverine^2+^ with a DNA molecule. Since many functional l-DNA constructs are beginning to emerge, structural elucidation of l-DNA isomers and their complexes with biomacromolecules at the atomic level will be important for understanding how mirror DNA can be integrated into biological systems (An *et al.*, 2020 ▸). Comparison of the positions of the potassium cations in the structures of left-handed Z-DNA with Put^2+^ and of right-handed Z-DNA with Cad^2+^ shows that despite the different chirality of the Z-DNA duplexes, the K^+^ ion has a preference for binding at OP_G6. Our studies of the inter­actions of Z-DNA with metal and biogenic polyamine cations confirm the notion that the ‘ionic atmosphere’ created by these positively charged species not only plays an important role in neutralizing the net charge of the nucleic acid, but also affects the structural stability of the DNA (Xiao *et al.*, 2014 ▸). It is remarkable that in four of the five metal complexes studied by us, the lability of the DNA molecules manifested itself as multiple conformations of the phosphate groups and even as double conformations of the bases (Zn^2+^, Cr^3+^, Mg^2+^ and K^+^ complexes; Drozdzal *et al.*, 2013 ▸, 2015 ▸, 2016 ▸, 2021 ▸). The above studies support the widely held opinion that from a chemical point of view, nucleic acid crystals are actually salts (or complexes) of metal ions. Thus, it is impossible to separate the behavior of these macromolecules from their interactions with metal cations (Kazakov & Hecht, 2006 ▸). In addition, double conformations of Z-DNA duplexes, often correlated with the presence of metals, may be important in mediating nucleic acid–protein interactions. To date, specific interactions of left-handed DNA with the Zα domain of six binding proteins have been reported (Bartas *et al.*, 2022 ▸).

Analysis of the torsion angles of the Cad^2+^ dication in this high-resolution structure, as well as in high-resolution structures of protein complexes with cadaverine (PDB entries 3qj5, 4ofg and 6ye7), indicates that in biological systems this simple diamine can adopt diverse conformations and consequently enter into a variety of interactions with biomacromolecular partners. Although the stabilization of nucleic acids by polyamine^*n*+^ cations has been studied for many years (Hou *et al.*, 2001 ▸), the structures that we have presented so far mostly show that the interaction of biogenic polyamine polycations with DNA is not entirely synonymous with the absence of alternate conformations of the DNA sugar-phosphate backbone. It was noted early on that the distances between the cationic centers of biogenic polyamines correspond well to the distances between the phosphate groups in DNA (Tsuboi, 1964 ▸; van Dam *et al.*, 2002 ▸). As illustrated by our structure, this assumption is especially true for Cad^2+^, where the N1^+^⋯N2^+^ distance of 7.451 (23) Å allows the formation of four hydrogen bonds to adjacent phosphate groups of the Z-DNA duplex (in the ideal Z-DNA hexamer, the P⋯P distances are in the range ∼5.69 to ∼7.18 Å). However, given the ability of polyamine^*n*+^ cations to adopt different conformations depending on the chemical environment in the crystal structures, it is reasonable to conclude that for both putrescine^2+^ and cadaverine^2+^ their (Z-)DNA binding site could change depending on the ionic conditions of the crystallization solution.

To summarize, the l-Z-DNA/Cad^2+^/K^+^, Z-DNA/Put^2+^/K^+^, Z-DNA/Spm^4+^/Zn^2+^ (Drozdzal *et al.*, 2013 ▸), Z-DNA/Mg^2+^, Z-DNA/Cr^3+^ (Drozdzal *et al.*, 2015 ▸) and Z-DNA-RNA/Ba^2+^ (Gilski *et al.*, 2016 ▸) structures support the statement that metal cations can modulate the DNA structure in a sequence-dependent manner by screening the repulsion between the negatively charged phosphates along the polymer, thus increasing its bending flexibility (Drozdetski *et al.*, 2016 ▸). Our results also confirm that mirror images of nucleic acids (‘spiegelmers’) are good candidates for studying the inter­actions in nucleic acid/metal^*m*+^/polyamine^*n*+^ complexes.

It is worth mentioning that not only mirror-image nucleotides can be used for the formation of right-handed Z-DNA. Satange *et al.* (2019 ▸) showed that the drug actinomycin D can tightly bind to G:G mismatched DNA duplexes through large-scale structural rearrangements, resulting in a right-handed Z-DNA-type structure (PDB entry 6j0h). It could be interesting to investigate whether other drugs (antibiotics) or ligands could cause similar distortions of nucleic acid fragments.

Finally, the picturesque story of the present refinement underlies the notion that high-quality diffraction data do not lie, and that by the thorough and accurate observance of state-of-the-art crystallographic practices one can even detect and rectify potential mishaps related to poor communication or to project transfer.

## 5. Data availability

The atomic coordinates and anisotropic ADPs, as well as the processed structure factors corresponding to the final model presented in this work, have been deposited in the PDB with accession code 8a71. The raw X-ray diffraction images, together with the complete *SHELXL* structure-factor input and output files, have been deposited in the Macromolecular Xtallography Raw Data Repository (MX-RDR) with DOI https://doi.org/10.18150/D5XSNJ.

## 6. Related literature

The following references are cited in the supporting information for this article: Dong (2003 ▸), Gao *et al.* (1993 ▸), Gessner *et al.* (1985 ▸) and Jean *et al.* (1993 ▸).

## Supplementary Material

PDB reference: right-handed Z-DNA containing 2′-deoxy-l-ribose in complex with cadaverine and K^+^, 8a71

Supplementary Tables. DOI: 10.1107/S2059798322011937/dw5233sup1.pdf

Raw diffraction images.: https://doi.org/10.18150/D5XSNJ

## Figures and Tables

**Table 1:** Data-collection and refinement statistics for L-d(CGCGCG)_2_/Cad^2+^/K^+^ Values in parentheses are for the outermost resolution shell.

Data collection	
Radiation source	P13, PETRA III, EMBL/DESY, Hamburg
Wavelength (Å)	0.7085
Temperature (K)	100
Space group	*P*2_1_2_1_2_1_
*a*, *b*, *c *(Å)	17.91, 31.13, 44.11
Resolution range (Å)	25.43–0.69 (0.74–0.69)
No. of reflections	73968†
Completeness (%)	96.3 (83.2)
Multiplicity	3.7 (1.2)
〈*I*/σ(*I*)〉	7.9 (2.0)
CC_1/2_ ‡ (%)	99.2 (78.6)
*R*_merge_ § (%)	8.1 (39.5)
Wilson *B* factor (Å^2^)	5.96
Refinement	
Refinement program	*SHELXL*
Resolution range (Å)	25.43–0.69
No. of reflections in working set	72088†
No. of reflections in test set	1880
*R*/*R*_free_ ¶ (%)	10.32/12.83
No. of atoms	
Nucleic acid	240
Solvent	121
Polyamine	7
Metal	1
〈*B*〉 (Å^2^)	
Nucleic acid chain *A*	5.20
Nucleic acid chain *B*	4.78
Solvent	12.76
Polyamine	9.48
Metal	4.73
R.m.s. deviations from ideal	
Bond lengths (Å)	0.012
Angles (°)	2.10

† Bijvoet pairs separated.

‡ Correlation between intensities from random half sets as defined by Karplus & Diederichs (2012 ▸).

§ *R*_merge_ = 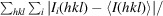 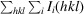 , where *I_i_* (*hkl*) is the intensity of observation *i* of reflection *hkl*.

¶ *R* = 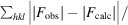 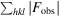 , where *F*_obs_ and *F*_calc_ are the observed and calculated structure factors, respectively. *R*_free_ was calculated analogously for the test reflections, which were randomly selected and excluded from the refinement.

**Table 2:** Coordination geometry around the metal ion in the L-d(CGCGCG)_2_/Cad^2+^/K^+^ structure Standard uncertainties are given in parentheses.

	Distances (Å)	Angles (°)						
	K^+^	W4	W11	W107(I)	W107(II)	OP1(II)_G6	O5′(II)_G6	OP1(II)_C9^i^
W4	2.491 (12)							
W11	2.769 (20)	75.7 (5)						
W107(I)	2.822 (60)	148.7 (9)	74.6 (9)					
W107(II)	3.017 (33)	174.2 (7)	101.0 (1)	26.8 (6)				
OP1(II)_G6	2.770 (13)	109.0 (4)	91.8 (7)	81.7 (8)	75.5 (5)			
O5′(II)_G6	2.987 (17)	74.4 (5)	117.1 (8)	129.0 (9)	111.4 (8)	50.4 (3)		
OP1(II)_C9^i^	3.005 (10)	84.2 (4)	154.5 (6)	120.8 (8)	98.0 (1)	109.7 (3)	70.9 (5)	
OP2(II)_C9^i^	2.679 (11)	91.6 (4)	111.7 (7)	90.3 (8)	85.3 (6)	152.3 (4)	123.1 (5)	52.7 (2)

Symmetry code: (i) −*x*, *y* + 1/2, −*z* + 1/2.
