# Supplementary material for: Right-handed Z-DNA at ultrahigh resolution: a tale of two hands and the power of the crystallographic method
Source: Acta Crystallogr D Struct Biol. 2023 Jan 20;79(Pt 2):133–9. doi: 10.1107/S2059798322011937 (PMC9912920; doi:10.1107/S2059798322011937)
Supplement: Supplementary file 1 [file d-79-00133-sup1.pdf]

Acta Cryst D

STRUCTURAL BIOLOGY

Volume 79 (2023)

Supporting information for article:

Right-handed Z-DNA at ultrahigh resolution: a tale of two hands and the power of the crystallographic method

Pawel Drozdzal, Tomasz Manszewski, Miroslaw Gilski, Krzysztof Brzezinski and Mariusz Jaskolski

Table S1. A selection of the crystal structures of nucleic acids in Z-form with metal cations and/or polyamines, deposited in the PDB or only described in the literature.

Sequence	Polyamine and/or metal cation(s)	Resolution (Å)	*R* (%)	Space group	PDB code or Reference
Structures with only polyamine cation(s)					
$d(CGCGCG)_2$	Spermine	0.55	7.77	$P2_12_12_1$	3p4j
$d(CGCGCG)_2$	Spermine	0.60	16.00	$P2_12_12_1$	1i0t
$d(CGCGCG)_2$	Spermine	1.00	18.50	$P2_12_12_1$	1d48
$d(CACGTG)_2$	Spermine / 13D[(a)]	1.20	18.30	$P2_12_12_1$	2f8w
$d(CGT^{2am}ACG)_2$	Spermine	1.35	17.40	$P3_221$	210d
$d(CGCGCG)_2$	Spermine	1.50	17.80	$P2_12_12_1$	1woe
$d(CGCGCG)_2$	PAW[(b)] (2x)	1.60	20.20	$P2_12_12_1$	2ie1
$d(CGCGCG)_2$	Spw[(c)]	1.80	22.20	$P2_12_12_1$	1v9g<br>(N-diffraction)
Structures with only metal cation(s)					

d(CGCGCG)$_2$	Mg^{2+} (2x)	0.78	13.90	*C*2/*c*	5jzq<br>(centrosymmetric)
d(CGCGCG)$_2$	Cr^{3+}	0.97	14.40	$P2_12_12_1$	4r15
(dCrGdCrGdCrG)$_2$	Ba^{2+}	1.09	11.36	$P2_1$	5ebi<br>(DNA-RNA chimera)
d(CGCGCG)$_2$	Mg^{2+} (4x)	1.00	17.50	$P2_12_12_1$	1dcg
d(CGCGCG)$_2$	Na^+/ Cu^{2+} (6x)	1.20	19.80	$P2_12_12_1$	1d39
d(CGCGCG)$_2$	Mg^{2+}/ $[Co(NH_3)_6]^{3+}$	1.25	18.00	$P2_12_12_1$	Gessner *et al.* (1985)
d(TGCGCA)$_2$	$[Co(NH_3)_6]^{3+}$	1.30	11.70	$P2_12_12_1$	362d
d(CGCGCG)$_2$	Ca^{2+}	1.30	19.50	$P3_221$	4fs6
d(^{m5}CGUA^{m5}CG)$_2$	Mg^{2+} (2x)	1.30	20.80	$P2_12_12_1$	1d41
d(^{m5}CGUA^{m5}CG)$_2$	Cu^{2+} (3x) / $CuCl_2$	1.30	20.90	$P2_12_12_1$	1d40
d(CGCGCG)$_2$	Mg^{2+}	1.30	21.30	$P3_2$	4fs5
d(C^{NH2}ACGTG)$_2$	Mg^{2+}	1.30	21.70	$P2_12_12_1$	1vty
d(aminohexyl-pCG^{Br}CGCG)	Ba^{2+}	1.40	16.40	*C*2	Jean *et al.* (1993)
d(CGCGCG)$_2$	Cu^{2+}	1.45	12.00	$P2_12_12_1$	4xsn
d[CGCG(DPY)AT(DRP)CGCG]$_2$ [(d)]	Cu^{2+}	1.50	16.90	$P2_1$	1jes
d(CGCG^{F}UG)$_2$	Mg^{2+}	1.50	17.20	$P2_12_12_1$	1dnf
d(CGCGTG)$_2$	Cu^{2+}	1.50	18.00	$P2_12_12_1$	Gao *et al.* (1993)
d(CGCGCG)$_2$	Co^{2+}/ Mg^{2+}	1.50	19.60	$P2_12_12_1$	Gao *et al.* (1993)
d(CGCGTG)$_2$	Mg^{2+}	1.50	20.00	$P2_12_12_1$	Gao *et al.* (1993)
d(pCGTACGTACG)$_2$	$[Co(NH_3)_6]^{3+}$	1.50	25.00	$P6_5$	1dn8
d(GCGCGA):d(TCGCGC)	$[Ru(NH_3)_6]^{3+}$	1.54	19.10	$P2_12_12_1$	2hto
d(CGCGCG)$_2$	Ca^{2+}/ Cu^{2+}	1.57	16.10	$P2_1$	5ihd
d(CACGCG):(CGCGTG)	Mn^{2+}	1.61	20.30	$P2_1$	4dwy

d(TGCGCA)$_2$	Mg^{2+} (2x)	1.64	21.20	$P2_12_12_1$	1ljx
d(GCGCGCG)$_2$	$[Co(NH_3)_6]^{3+}$	1.65	18.40	$P2_12_12_1$	331d
d(CACGCG):(CGCGTG)	Ba^{2+}	1.67	18.90	$P2_12_12_1$	4e2r
d(CACGCG):(CGCGTG)	Ba^{2+}	1.67	21.70	$P2_12_12_1$	3fqb
d(^{m5}GCGCGCG)$_2$	Mg^{2+}/ $[Co(NH_3)_6]^{3+}$	1.68	20.70	$P2_12_12_1$	313d
d(TCGCGCG)$_2$	Mn^{2+}	1.70	17.30	$C2$	4kmf<br>(Z-DNA/protein complex)
d(CGCGCG)$_2$	Rb^{+} (3x)	1.70	17.50	$P2_12_12_1$	Dong (2003)
d(CGCGCA):d(TGCGCG)	$[Co(NH_3)_6]^{3+}$	1.71	21.00	$P2_12_12_1$	1xa2
d(CACGCG):(CGCGTG)	Ba^{2+}	1.72	20.40	$P2_1$	4e4o
d(CACGCG):(CGCGTG)	Mn^{2+}	1.76	22.70	$P6_5$	4dy8
d(GCGCGCG):d(CCGCGCG)	Mg^{2+}/ $[Co(NH_3)_6]^{3+}$	1.80	20.90	$P2_12_12_1$	312d
d(CACGCG):(CGCGTG)	Ba^{2+}	1.86	21.60	$P6_5$	4e60
d(CGCGCA):d(TGCGCG)	$[Co(NH_3)_6]^{3+}$	1.86	27.90	$P6_5$	1xam
d(GCGCGCG):d(TCGCGCG)	Mg^{2+}/ $[Co(NH_3)_6]^{3+}$	1.90	19.10	$P2_12_12_1$	314d
d(CGCGCG)$_2$	Ca^{2+}/ Cu^{2+}	2.15	23.10	$P2_1$	4xqz
d(CGTGCG)$_2$	Mn^{2+}	2.15	26.90	$C222_1$	3g2r
d(^{Br}UGCGCG)$_2$	Mg^{2+}	2.25	15.60	$P2_12_12_1$	1da1
(dU)r(CGCGCG)$_2$	Na^{+}	2.25	19.90	$C222_1$	2gxb<br>(Z-RNA/protein complex)
d(TGCGCG):d(CGCGCA)	$[Ru(NH_3)_6]^{3+}$	2.60	26.70	$P6_5$	2htt
d(CACGCG):(CGCGTG)	Mn^{2+}	2.80	16.70	$P2_1$	3fq5
d(pCGCGCG)$_2$	Mg^{2+}	3.00	18.20	$C222_1$	392d
Structures with polyamine and metal cations					
d(CGCGCG)$_2$	K^{+}/ Putrescine^{2+}	0.60	8.77	$P2_12_12_1$	7atg
L-d(CGCGCG)$_2$	K^{+}/ Cadaverine^{2+}	0.69	10.54	$P2_12_12_1$	8a71<br>(right-handed Z-DNA, this work)

$d(CGCGCG)_2$	Mn^{2+}/ Spermine	0.75	7.09	$P2_12_12_1$	4hig
$d(CGCGCG)_2$	Zn^{2+} (2x) / Spermine	0.85	9.22	$P2_12_12_1$	4hif
$d(CGCGCG)_2$	Mg^{2+}/ Spermine (2x)	0.90	14.00	$P2_12_12_1$	2dcg
$d(CGCGCG)_2$	Mg^{2+}/ Spermine	0.95	8.60	$P2_12_12_1$	1ick
$d(CGCGCG)_2$	Mg^{2+}/ Trientine^{4+}[(e)] (2x)	1.00	16.50	$P2_12_12_1$	1dj6
$d(CGCGCG)_2$	Na^+/ Mg^{2+} (3x)/ P24[(f)]	1.00	16.10	$P2_12_12_1$	292d
$d(CGCGCG)_2$	Na^+/ Spermine	1.00	18.00	$P2_12_12_1$	131d
$d(CGCGCG)_2$	Mg^{2+} (2x)/ Thermospermine^{4+} [(g)] (2x)	1.00	19.00	$P2_12_12_1$	336d
$d(CGCGCG)_2$	Na^+/ Mg^{2+} (3x)/ Spermidine	1.00	19.10	$P2_12_12_1$	293d
$d(CGCGCG)_2$	Na^+/ Mg^{2+} (3x)/ Spermidine	1.00	24.40	$P2_12_12_1$	2elg
$d(Cp_{Se}GCGCG)_2$	Mg^{2+}/ Spermine	1.10	9.70	$P2_12_12_1$	1vro
$d(CGCGCG)_2$	Ca^{2+} / Spermidine	1.45	12.10	$P2_12_12_1$	6bst
$d(CGCGCG)_2$	Co^{2+}/ Spermine^{4+}	1.50	23.70	$P2_12_12_1$	Gao *et al.* (1993)
$d(CGT^{2am}ACG)_2$	$[Pt(NH_3)_3]^{2+}$/ Spermine^{4+}	1.60	17.00	$P3_221$	211d

[a] 1,3-diaminopropane

[b] N-(2-aminoethyl)-N'-{2-[(2-aminoethyl)amino]ethyl}ethane-1,2-diamine

[c] Spermine^{4+} in fully deuterated form

[d] The N3 atoms are in the aromatic rings of modified nucleotides containing pyridine (DRP) and pyridine-2,6-dicarboxylic acid (DPY)

[e] N,N'-bis(2-aminoethyl)-1,2-ethanediamine

[f] 1-(aminoethyl)amino-4-aminobutane

[g] N-(3-amino-propyl)-N-(5-aminopropyl)-1,4-diaminobutane

Table S2. Sugar and phosphate backbone torsion angles, calculated by the *3DNA* program (Lu & Olson, 2003), as well as the furanose pseudorotation amplitude (τ_m) and phase angle of pseudorotation (P), calculated by the method of Jaskolski (1984). All numerical x/y values in the table are in degrees, and were calculated (x) for the left-handed model of D-Z-DNA, PDB ID 3p4j, and (y) the right-handed model of L-Z-DNA (this work). Because of the inverted configuration, the P values for 2'-deoxy-L-ribose are shifted on the pseudorotation wheel by 180°.

**Angle**	**C1**	**C3**	**C5**	**C7**	**C9**	**C11**	**G2**	**G4**	**G6**	**G8**	**G10**	**G12**
α	-	-148.1 / 147.9	167.9 / -163.9	-	-142.6 / 145.3	-148.9 / 151.3	60.2 / -63.9	68.7 / -74.6	75.2 / -90.6	61.1 / -62.6	65.2 / -67.4	78.5 / -79.3
β	-	-121.3 / 118.2	167.1 / -162.7	-	132.1 / 126.1	-122.8 / 121.7	-169.3 / 170.3	-170.2 / 175.6	-179.4 / 176.0	-172.6 / 174.1	-179.5 / 177.1	-176.3 / 176.3
γ	53.3 / -55.0	54.5 / -52.2	44.1 / -52.9	54.2 / -55.4	56.3 / -57.2	54.9 / -55.3	178.2 / -177.5	-179.5 / -177.2	-176.4 / 178.3	176.8 / -175.8	179.8 / -179.6	-175.2 / 175.9
δ	142.7 / -141.9	148.2 / -149.0	142.9 / -146.1	148.4 / -146.0	146.7 / -144.6	141.2 / -140.5	90.6 / -89.9	92.4 /-94.4	147.5 / -142.8	92.9 / -96.8	98.5 / -95.3	152.8 / -152.6
ε	-92.8 / 93.2	-98.4 / 96.7	-94.5 / 95.3	-88.0 / 90.8	-91.6 / 92.5	-96.8 / 97.0	-120.2 / 119.6	-177.9 / -179.3	-	-111.1 / 114.9	-117.3 / 116.2	-
ζ	78.0 / -73.5	71.8 / -69.2	73.5 / -60.2	73.5 / -75.1	74.0 / -71.4	67.3 / -66.1	-68.9 / 69.9	65.6 / -62.7	-	-77.5 / 79.2	-69.2 / 70.1	-
χ	-152.3 / 153.0	-154.4 / 154.0	-143.6 / 148.7	-145.1 / 147.2	-155.3 / 155.0	-156.0 / 154.5	59.2 / -58.8	55.8 / -58.1	77.2 / -77.1	67.3 / -64.8	62.1 / -60.0	77.7 / -77.5
ν_0	-25.1 / 25.1	-25.5 / 25.2	-22.7 / 25.3	-20.1 / 23.4	-21.3 / 21.9	-24.7 / 25.3	-6.5 / 7.5	-4.1 / 3.5	-19.4 / 18.5	-8.0 / 9.0	-0.1 / 1.7	-19.7 / 20.9
ν_1	35.4/ -35.0	38.4 / -38.8	33.3 / -36.3	33.6 / -35.1	34.4 / -34.5	35.0 / -35.1	-13.6 / 13.5	-13.6 / 14.2	33.1 / -31.7	-11.4 / 11.5	-15.3 / 16.0	36.1 / -37.3
ν_2	-31.7 / 31.2	-36.2 / 37.7	-30.8 / 33.0	-33.8 / 33.0	-33.7 / 33.4	-31.7 / 31.5	26.7 / -27.4	24.7 / -24.9	-33.4 / 32.1	24.8 / -25.6	23.5 / -25.9	-37.8 / 38.0
ν_3	18.1 / -17.2	22.6 / -24.0	18.4 / -19.3	23.2 / -20.6	22.1 / -21.5	18.2 / -17.8	-31.2 / 32.8	-27.7 / 27.5	22.9 / -22.2	-29.9 /31.3	-23.9 / 27.6	27.7 / -26.7
ν_4	4.3 / -4.8	1.8 / -0.4	2.5 / -3.6	-2.1 / -1.8	-0.7 / 0.0	4.1 / -4.5	24.0 / -25.8	20.2 / -19.8	-2.4 / 2.4	24.0 / -25.6	15.3 / -18.8	-5.1 / 3.9
**Pseudorotation parameters**												
P	154.9(7)/154.0(6)	159.2(7)/161.0(7)	157.5(7)/156.1(7)	165.0(7)/159.0(8)	162.7(6)/161.6(7)	155.3(7)/154.5(8)	29.9(8)/31.4(9)	26.5(7)/25.2(8)	165.5(6)/165.7(6)	33.2(7)/34.3(8)	18.2(7)/21.6(8)	169.0(7)/167.1(7)
τ_m	36.0(5)/35.5(4)	39.7(5)/40.7(5)	34.2(4)/37.0(5)	35.9(4)/36.3(5)	36.1(4)/36.0(4)	35.7(4)/35.8(5)	31.5(4)/33.0(5)	28.2(3)/28.2(4)	35.3(4)/33.9(4)	30.2(4)/31.6(4)	25.2(3)/28.6(4)	39.6(5)/40.0(5)
**Sugar pucker**	C2'-*endo*	C2'-*endo*	C2'-*endo*	C2'-*endo*	C2'-*endo*	C2'-*endo*	C3'-*endo*	C3'-*endo*	C2'-*endo*	C3'-*endo*	C3'-*endo*	C2'-*endo*

References

Dong, X. (2003). *Acta Cryst.* D**59**, 1336-1338.

Gao, Y. -G., Sriram, M. & Wang, A. H. -J. (1993). *Nucleic Acids Res.* **21**, 4093-4101.

Gessner, R. V., Quigley, G. J., Wang, A. H., van der Marel, G. A., van Boom, J. H. & Rich, A. (1985). *Biochemistry* **24**, 237-240.

Jaskolski, M. (1984). *Acta Cryst.* A**40**, 364–366.

Jean, Y.-C., Gao, Y.-G. & Wang, A. H.-J. (1993). *Biochemistry* **32**, 381–388.

Lu, X. J. & Olson, W. K. (2003). *Nucleic Acids Res*. **31**, 5108–5121.
